# The "Doctor's" Dictum Questioned

**Published:** 1889-07-13

**Authors:** A. M. F. Cole

**Affiliations:** (District Nurse). Hyde, Cheshire


### Editor's Letter-Box.

Our Correspondents are reminded that prolixity is a great bar to publication, and that brevity of style and conciseness of statement greatly facilitate early insertion.]

### "THE DOCTOR'S" DICTUM QUESTIONED.

Sir,—The Doctor asserts many things about the evils of excess in religion from his pulpit this week; amongst others: (1) That because we are "mortal, material, and human," we should follow the "moderate rule of Nature" in our religion.

Does the Doctor refer to the Christian religion? Our Founder did not teach or practise thus. He fasted, prayed, taught, kept vigil, and suffered death through "excess" of religion. His disciples followed His example. From St. Paul's account of himself it would appear that he was a notable example of such excess. Self-preservation is the first law of Nature, and self-sacrifice the first law of the Christian religion: how then can the Doctor teach us that the same laws govern each?

(2) That the multiplication of church-goings, undue fastings, and mortifications result in mental feebleness and bodily disease—in "an excitable, irrational, useless, and injurious fanatic."

Does this description apply to General Gordon, or to the present Pope Leo? Yet both have been guilty of great "excess" in religion. The writings of such "fanatics" as St. Augustine, St. Thomas Aquinas, St. Thomas à Kempis, Cardinal Newman, and many Jesuits of to-day exhibit no symptoms of mental feebleness or an irrational mind, and from what we read or see of these men we can find little evidence of those mental or bodily afflictions mentioned by the Doctor. I think all who have had most intercourse with those who do indeed multiply prayers, fasting, and mortifications—the priests, Jesuits, and monks of the Catholic Church—will allow that, be it the point of a joke, an abstract mental calculation, or a scalded foot, they are seldom wanting in the appreciation, the solution, or the remedy required for the occasion. According to the Doctor they ought to be "mentally feeble, bodily diseased, excitable, irrational, useless, and injurious fanatics!"

(3) "It would be more than strange if reason, which is the perfection and safety of all other things, should be both useless and injurious in religion."

Was the incarnation of perfection unreasonable? Is it not more reasonable to obey what is revealed to us by His teaching and example, than to attempt to make human reason the judge as to how far we shall follow the Divine example? "Reason never shows herself more reasonable than in this—to cease reasoning upon things beyond reason." Reason leads us to Faith, but Faith goes beyond, yet never contradicts, Reason.

Perhaps the Doctor is a Pantheist or Deist, and so argues justly according to his own lights. But many a young nurse may mistake for Christian maxims these warnings against that "excess of religion" which Our Master lived and died to vindicate, or, I may say, to inculcate. I do not think our nursing would be improved if we took our cue from the Doctor's sermon, and made Nature's primary law—self-preservation—our motto, rather than Our Lord's law—self-sacrifice. "Inasmuch as ye do it unto one of the least of these, My brethren, ye do it unto Me," and "The measure of love is love's *sacrifice*." Let *these* be our maxims in our religion and in our work, and no "moderate laws of Nature." A. M. F. Cole (District Nurse).

Hyde, Cheshire, July 3, 1889.

[Miss Cole has somewhat misunderstood the teaching of the Doctor's Pulpit; and the Doctor, who writes from the Christian standpoint, thinks she also misunderstands the teaching of the New Testament on the subject.—Ed.]

